# Biphasic parapharyngeal synovial sarcoma: a cytologic and immunocytologic report of a case

**DOI:** 10.1186/1742-6413-3-20

**Published:** 2006-08-14

**Authors:** Bijan Khademi, Yahya Daneshbod, Shahrzad Negahban, Khosrow Daneshbod, Massud Kaviani, Mohammad Mohammadianpanah, Mohammad J Ashraf

**Affiliations:** 1 Department of Head and Neck Surgery, Khalili Hospital, Shiraz Medical School, Shiraz, Iran; 2 Department of Cytopathology, Dr. Daneshbod Pathology Laboratory, Shiraz, Iran; 3 Department of Hematopathology, Dr. Daneshbod Pathology Laboratory, Shiraz, Iran; 4 Department of Surgical Pathology, Dr. Daneshbod Pathology Laboratory, Shiraz, Iran; 5 Department of Radiation-Oncology, Nemazee Hospital, Shiraz University of Medical Sciences, Shiraz, Iran; 6 Department of Pathology, Khalili Hospital, Shiraz Medical School, Shiraz, Iran

## Abstract

**Background:**

Synovial sarcoma is a rare soft tissue sarcoma in the head and neck region and parapharyngeal space. There is no previous cytologic report of synovial sarcoma of parapharynx. The cytologic and immunocytochemical findings of a parapharyngeal biphasic synovial sarcoma together with diagnostic pitfalls are described.

**Case report:**

A 21-year-old girl presented with a 6-month history of progressive right arm pain, neck mass and upper aerodigestive tract obstruction. On physical examination there was a large painless mass arising from the right-sided parapharyngeal space causing airway obstruction. Initial magnetic resonance imaging (MRI) revealed a large tumor in the right-sided parapharyngeal space. Fine needle aspiration through cervical region was performed and was reported as benign spindle cell tumor. Smears were cellular and composed mostly of tight and loose clusters of spindle cells. Epitheloid cells could also be identified intermingled with them. She underwent near total resection of the tumor. Pathologic report disclosed the diagnosis of synovial sarcoma. She then received postoperative adjuvant external radiotherapy.

**Conclusion:**

Due to rarity of this tumor in this region and nonspecific cytologic features, we could not differentiate this tumor from the other more common spindle cell neoplasms. Considering synovial sarcoma in this region and immunocytochemistry can be helpful in rendering a correct initial diagnosis of this tumor.

## Background

Synovial sarcoma(SS) usually occurs in young adults and is found in the paraarticular areas of the tendon sheaths and joints in the lower and upper-extremity.

The surgical pathology diagnosis can be especially challenging when SS arises in unusual locations, young patients, or have a poorly differentiated morphology[1,2].

Salivary gland and neurogenic tumors are the most common neoplasms involving the parapharyngeal space[3]. SS accounts only 3–10% of all head and neck soft tissue sarcomas[4,5]. The cytology of SS has been well described in the literature[1,2,6-8]. However there is no cytologic report of this tumor in head and neck region. Herein we describe a case of parapharyngeal SS.

## Case presentation

**Figure 1:**
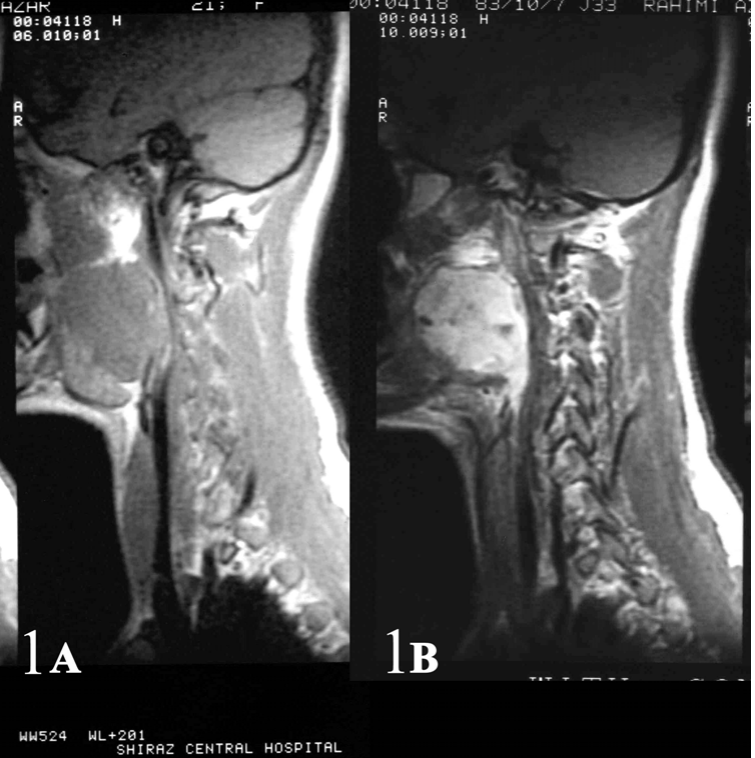
AB: Sagital and coronal (2AB) contrast-enhanced MR images of the right parapharyngeal synovial sarcoma.

**Figure 2:**
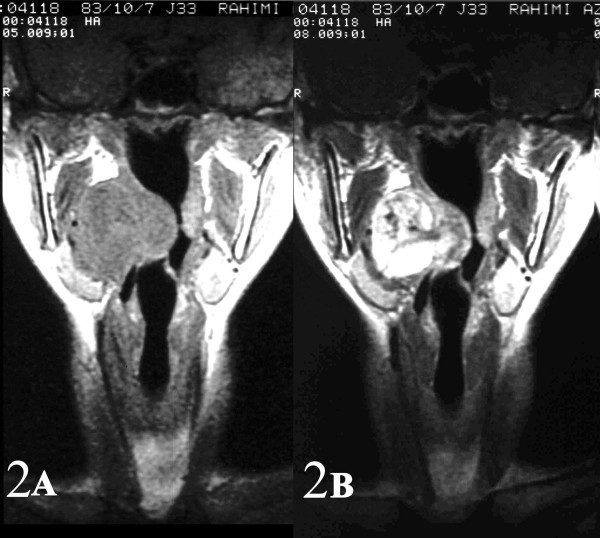
AB: Sagital and coronal (2AB) contrast-enhanced MR images of the right parapharyngeal synovial sarcoma.

A 21-year-old girl with no significant past medical history, presented with a 6-month history of progressive right arm pain, followed by a 3-month history of right-sided otalgia, jaw and shoulder pain, dysphagia, dyspnea and cervical mass. On physical examination, she had a large painless mass arising from the right-sided parapharyngeal space causing severe airway narrowing with no cervical lymphadenopathy. Initial magnetic resonance imaging (MRI) revealed a large contrast-enhanced mass arising from the right-sided parapharyngeal space causing airway obstruction and pressure effect on the major cervical vessels(Fig 1, 2). Fine needle aspiration through cervical region was performed and reported as benign spindle cell tumor. The patient then underwent near total tumor resection. In less than 8 months after the completion of multi-modality therapy, she developed multiple lung metastases.

### Cytology

The FNA biopsy was performed using 25 gauge 1-inch needle attached to a disposable 20-mm syringe. Approximately one half of the smears were immediately wet-fixed with 95% ethyl alcohol for Papanicolaou and immunostaining. The remaining smears were air-dried and stained by Wright method.

### Immunocytochemistry and immunohistochemistry

Immunohistochemical stains were performed on prefixed unstained cytologic smears and paraffin embedded tissue by the standard avidin-biotin technique after a microwave citrate buffer antigen retrieval step. The panel of antibodies used included cytokeratin ([CK]AE1/AE3, 1:80; Dako Co.), S-100 protein (1:400; Dako), muscle specific actin (1:10; BioGenix, San Ramon, CA), desmin (1:100; Dako), actin (1:100; Dako), Vimentin (1:100;Dako), and epithelial membrane antigen ([EMA], 1:300; Dako).

### Cytologic findings

**Figure 3:**
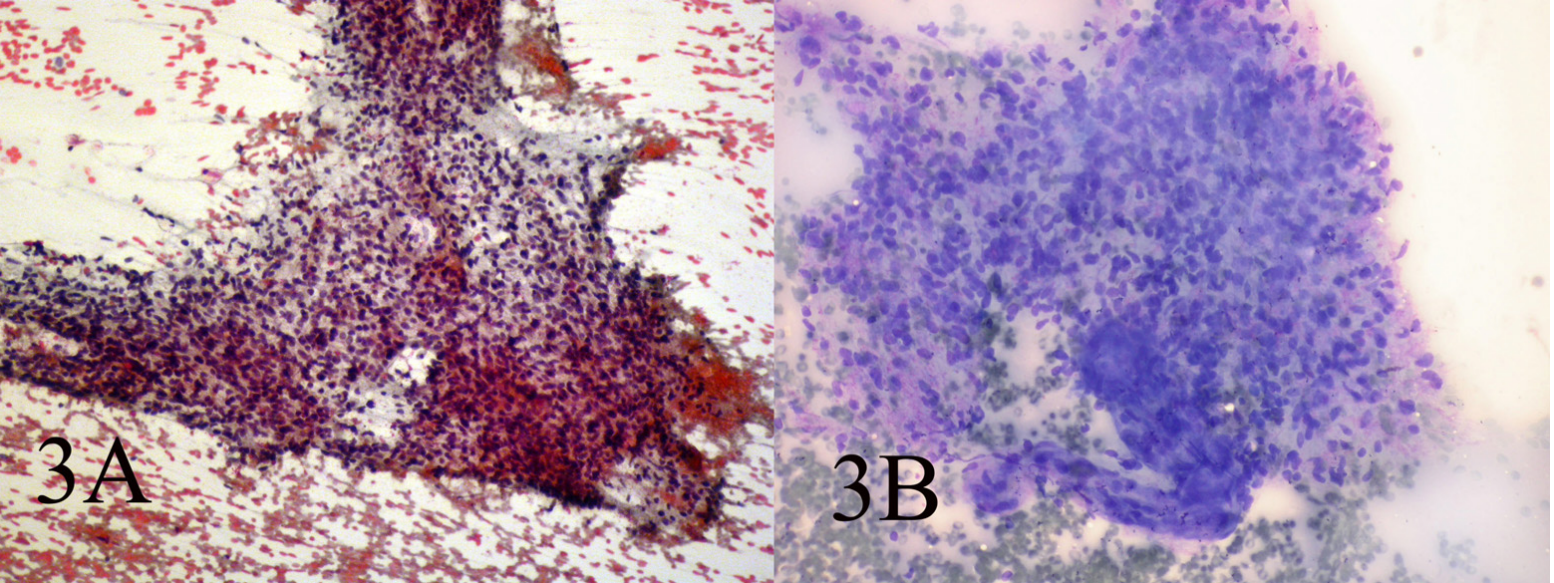
AB: Small and large clusters of cells with bland chromatin, inconspicuous nucleoli, oval to spindle-shaped cytoplasm and branching tumor tissue fragments and vessel stalks(Papanicolaou 100, Wright 100).

**Figure 4:**
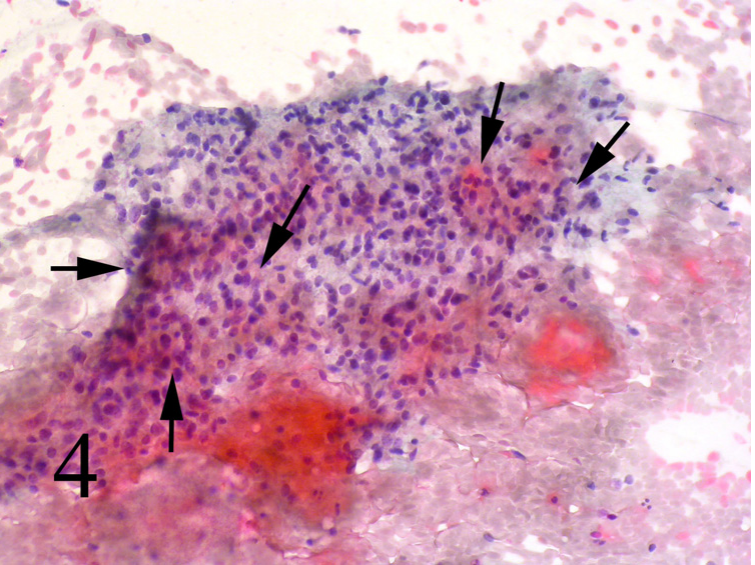
Acinar structures scattered in between spindle cells with scant mucin Background (Papanicolaou, 100).

**Figure 5:**
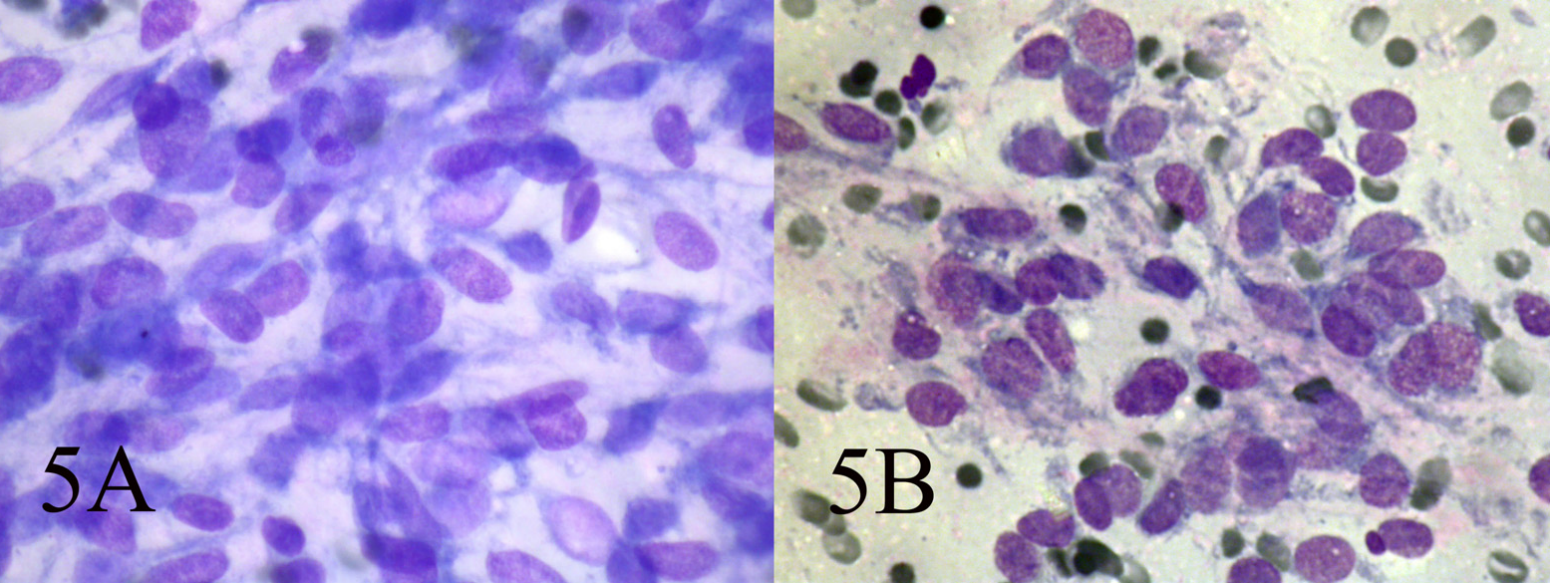
AB: Ovoid cells have short and long cytoplasmic processes; and finely granular chromatin and small or indistinct nuclei (Wright, Wright 400, 400).

**Figure 6:**
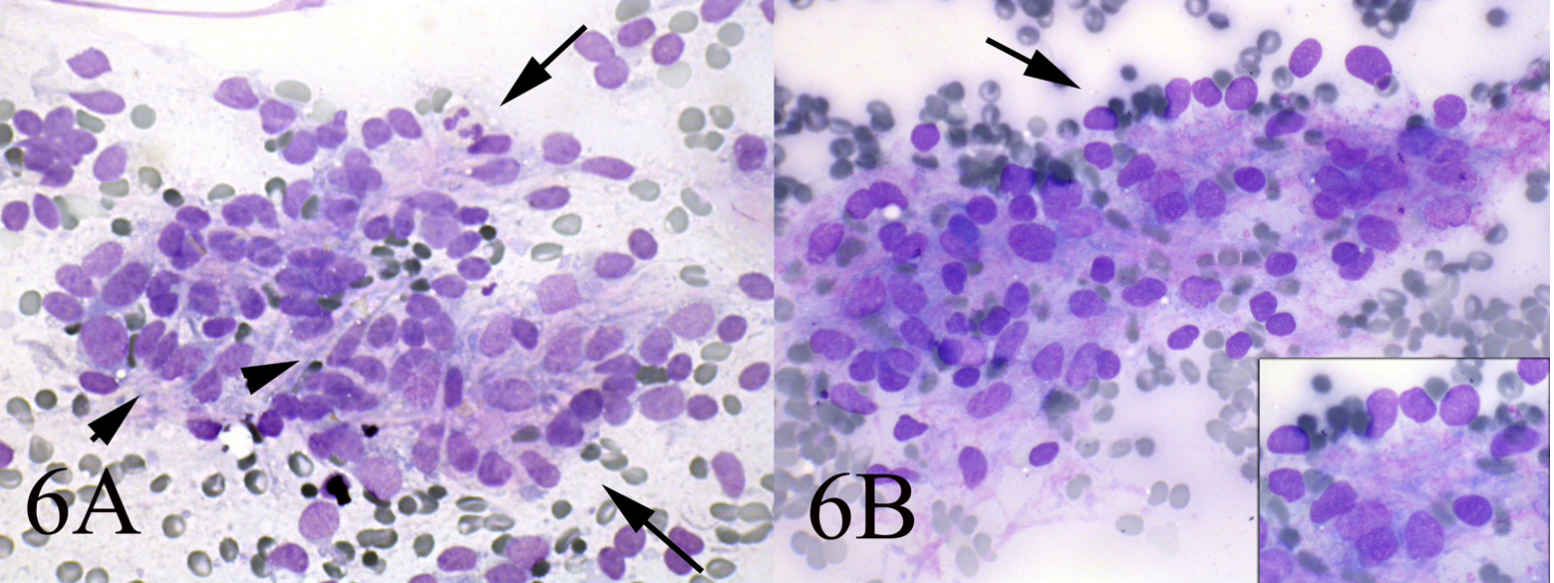
AB: Smears also showed a mixture of dispersed spindle cells(arrow-head) and epitheloid cells which occasionally formed acinar structures(long arrow) (Wright, Wright 400, 400).

**Figure 7:**
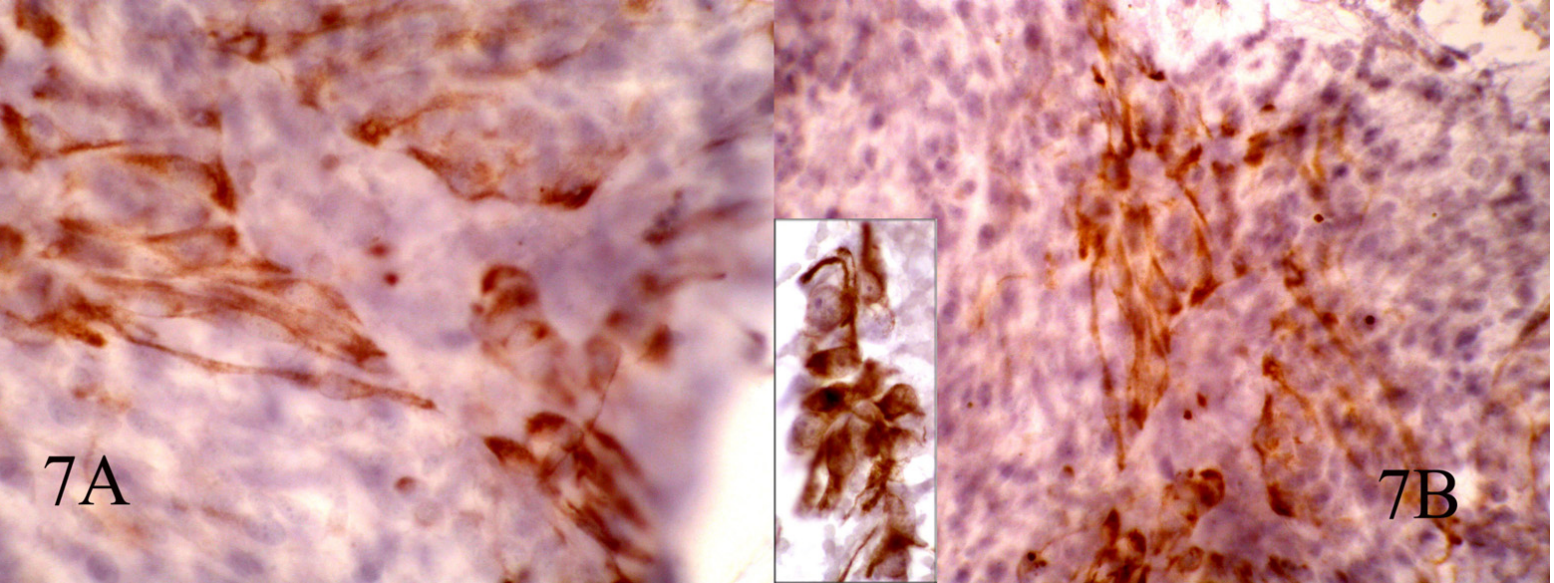
AB: Immunocytochemical staining for CK show focal positivity in spindle cells and also positive EMA in epitheloid cells(inset). (400, 400, inset:600)

Small and large clusters of cells with bland chromatin, inconspicuous nucleoli, oval to spindle-shaped cytoplasm with branching tumor tissue fragments(Fig. 3A), and vessel stalks were identified (Fig. 3B). Acinar structures scattered in between spindle cells with scant mucin background were also seen(Fig. 4). Ovoid cells had short and long cytoplasmic processes; and finely granular chromatin and small or indistinct nuclei(Fig. 5AB). Smears also showed a mixture of dispersed or clusters of spindle cells with bland chromatin, inconspicuous nucleoli, and epitheloid cells which occasionally formed acinar structures(Fig. 6AB). Immunocytochemical staining for CK showed focal positivity in the spindle cell component(Fig. 7A) and also positivity in epitheloid cells for EMA (Fig. 7B). Immunocytochemical staining for S100, actin and desmin were negative.

### Histopathology

**Figure 8:**
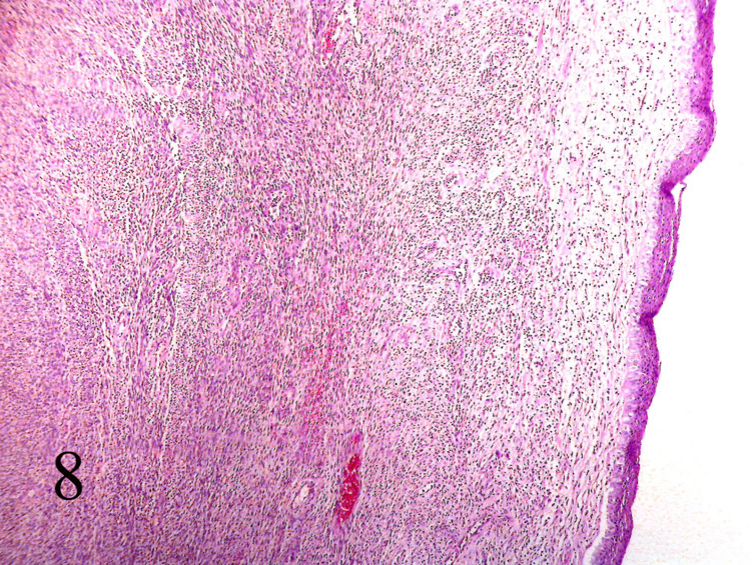
Section of parapharynx showed a submucosal hypercellular tumoral tissue. (Hematoxilin & eosin, 100).

**Figure 9:**
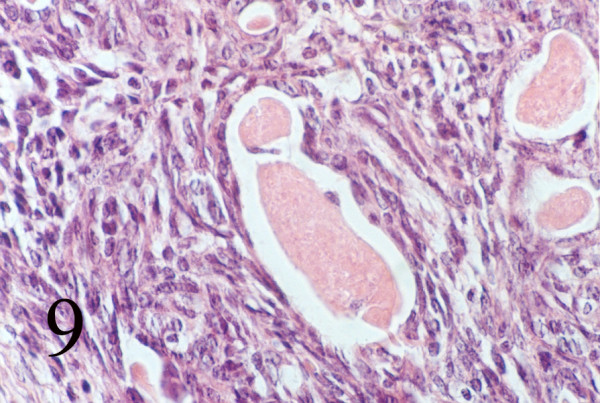
Biphasic pattern of glands and spindle cells (Hematoxilin & eosin, 400).

**Figure 10:**
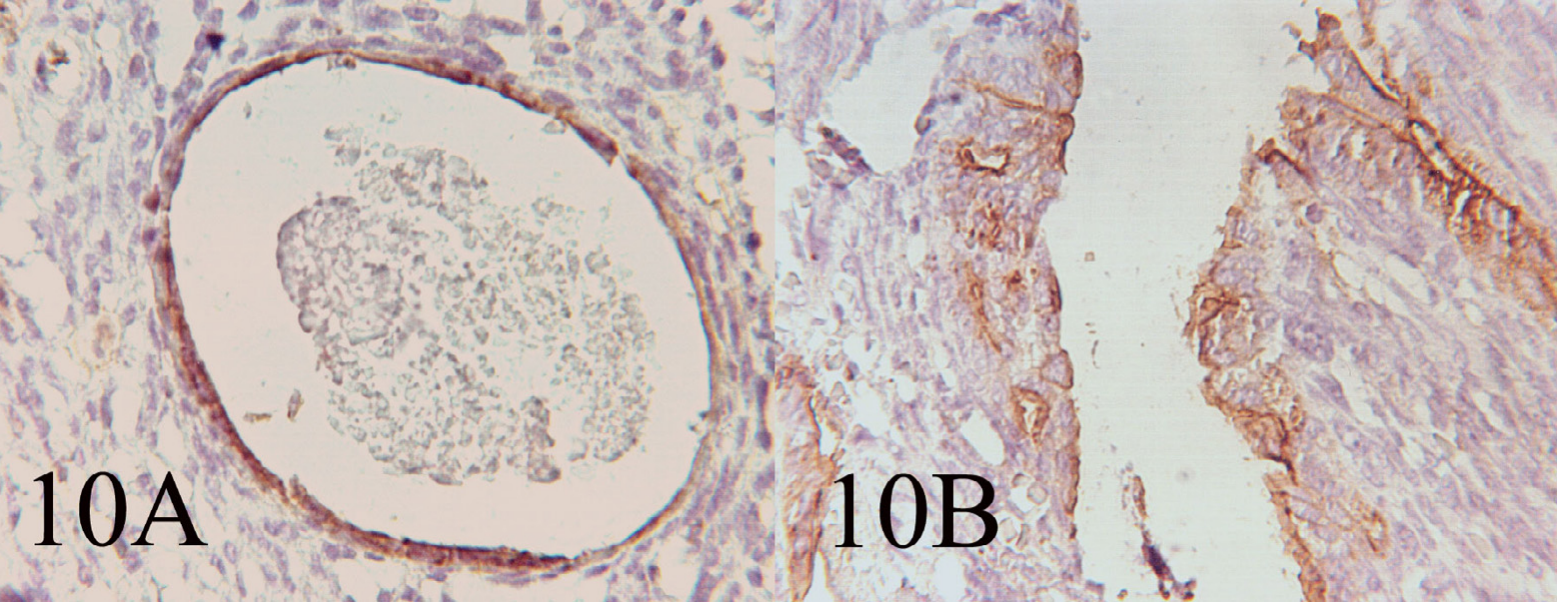
AB: Immunohistochemistry showing EMA and Cytokeratin positivity of the cells lining the glandular and cystic spaces. (400, 400)

Grossly a fragile, spongy, nonencapsulated 4 × 3 × 2 cm mass was resected. Microscopic examination showed submucosal hypercellular tumoral tissue (Fig. 8). On higher magnification the tumor had a biphasic pattern(Fig. 9). Epithelial cells formed glands that contained eosinophilic secretions. The stromal component was composed of oval to spindle cell arranged as sheets and fascicles. Immunohistochemical study revealed cytokeratin and EMA positively in the epithelial component(Fig. 10AB) and diffuse vimetin positivity in spindle cells.

## Discussion

Parapharyngeal space, a potential fascial plane of the head and neck, may be involved by neoplastic disease which represents less than 1% of all head and neck tumors. 80% of parapharyngeal neoplasms are benign and 20% are malignant[9]. Benign salivary gland and neurogenic tumors are the most common neoplasms involving the parapharyngeal space and sarcomas represent a minority of these neoplasms[3]. SS of the head and neck region are extremely rare, accounting for only less than 10% of all head and neck soft tissue sarcomas. This soft tissue neoplasm generally does not originate from synovial tissue and probably originates from the pluripotent mesenchymal cells[10]. About 100 cases of the SS of the head and neck region have been reported in the literature[10]. These patients typically present with a painless mass in the third and fourth decades of life. Males tend to be affected more than females.

Microscopically, the classic form of the synovial sarcoma has biphasic pattern composed of two cell population: epithelial cells and spindle cells. Epithelial cells form nests and glands; line slit like spaces and have abundant eosinophilic cytoplasm, distinct cell border and eosinophilic nuclei. The glands contain eosinophilic PAS-positive secretions. Another component is sheets and fascicles of cells with storiform, herring bone patterns or random arrangement. The cells have scant pale cytoplasm and oval to spindle shaped hyperchromatic nuclei[6,11].

Special immunohistochemical stains and cytogenetic studies can help in confirming the diagnosis. Immunohistochemically, there is strong reactivity for keratin in epithelial areas and often in spindle cells as well. Synovial sarcoma are also positive for EMA, vimentin, BCL-2, CD99 and calponin. A specific translocation between chromosome X and 18, t(x; 18) (p11.2; q11.2) is mandatory for a conclusive diagnosis of synovial sarcoma[6,10,11]. The detection of this characteristic chromosomal translocation is useful in confirming the diagnosis of synovial sarcoma, particularly when histologic studies are ambiguous.

The diagnostic cytologic criteria of SS have previously been described. The following features have been described to be characteristic of SS: smears are cell-rich, stroma-poor, of striking uniformity, lacking nuclear pleomorphism, and composed mostly of ovoid to rounded tumor cells with scant tapering cytoplasm. Branching papillary-like tumor tissue fragments with vessel stalks, acinar-like structures, and comma-like nuclei are also characteristic of both monophasic and biphasic variants[2,6-8,11]. Kilpatrick et al. have suggested that the presence of epithelial cells is necessary for the diagnosis[1], a finding rarely present, as confirmed in later reports[8]. Åkerman et al. reported epithelial cells in only one of 25 cases[11], Kilpatrick et al.[1] in two of 13, Viguer et al. [12]in one of 12 and Klijanienko et al[8] in three of the 11 cases. By contrast, Ryan et al. described epithelial cells in four of five cases[13]. Whether the presence of an epithelial component is necessary for the accurate cytological diagnosis of SS is debatable.

Our findings support previous studies[1,8,11,12] when we found epithelial areas only at retrospective evaluation of the slides and later confrmed by immunocytochemistry. However due to the rarity of this tumor in this location and the benign appearing cytomorphology we reported the case as a benign spindle cell tumur, consistent with neural origin.

The presence of a classical pattern is highly suggestive of SS and the presence of epithelial cells is mandatory. As in surgical pathology, entities to include in the differential diagnosis of SS are: fibrosarcoma, malignant hemangiopericytoma (MHPC), and malignant peripheral nerve sheath tumor (MPNST). Ewing/peripheral neuroectodermal tumor (pPNET) and malignant melanoma should also be differentiated from poorly differentiated SS. Exact typing in spindle-cell mesenchymal tumors is considered of little clinical importance since therapeutic management is often identical. Fibrosarcoma, MHPC, and MPNST may have similar morphology as SS. In the absence of the classical pattern, or epithelial cells, the diagnosis of SS may be difficult and may require ancillary techniques such as immunocytochemistry, cell blocks, genetic studies, or electron microscopy[1,2,7,8,11,14,15].

As in our case focal positivity for EMA in spindle cell component and positivity for CK in epitheloid cells is not seen in any of the mentioned differential diagnosis. In the absence of a precise diagnosis, surgical biopsy is indicated. Differential diagnostic parameters like vascular pattern in MHPC, nuclear palisading in MPNST, collagenosis or intersecting fascicles in fibrosarcoma may be present in synovial sarcoma but are more in the domain of histopathology than cytopathology. Fibrosarcoma and MHPC remain a diagnosis of exclusion. There are tumors that show an overlapping morphology with SS, due to the presence of numerous spindle-shaped cells without nuclear pleomorphism[6,8]. In MPNST a "wavy appearance," elongated and slender nuclei, focal pronounced nuclear atypia, and fibrillary metachromic stroma are indicative of the diagnosis. However, other features such as the location in the vicinity of a nerve, a history of neuroma or von Recklinghausen's disease may help in the differential diagnosis. Distinctive immunocytochemical analysis may be inconclusive, since 30% of SS may show unexpected S-100 protein positivity and only 50% of MPNST express S-100 protein[6,7]. However positivity for EMA and CK helps for a correct diagnosis.

This potential for misdiagnosis is accentuated when SS occur in unusual locations and in a young age group, as demonstrated in our case. Helpful confirmatory studies for the diagnosis of SS includes positive immunoperoxidase staining for EMA and cytokeratin, or the presence of the characteristic translocation (X; 18). In summary we would like to emphasize the importance of ancillary techniques such as immunocytochemistry and cytogenetics on aspiration material, besides conventional methods of rapid stains for a cytopathologic diagnosis of SS.

## References

[B1] Kilpatrick SE, Teot LA, Stanley MW, Ward WG, Savage PD, Geisinger KR (1996). Fine-needle aspiration biopsy of synovial sarcoma. A cytomorphologic analysis of primary, recurrent, and metastatic tumors. Am J Clin Pathol.

[B2] Silverman JF, Landreneau RJ, Sturgis CD, Raab SS, Fox KR, Jasnosz KM, Dabbs DJ (2000). Small-cell variant of synovial sarcoma: fine-needle aspiration with ancillary features and potential diagnostic pitfalls. Diagn Cytopathol.

[B3] Hughes KV, Olsen KD, McCaffrey TV (1995). Parapharyngeal space neoplasms. Head Neck.

[B4] Agada FrankO, Justin Murphy, Ravi Sharma (2005). Biphasic synovial sarcoma of the posterior pharyngeal wall: A case report. Ear, Nose & Throat Journal.

[B5] Pai S, Chinoy RF, Pradhan SA (1993). Head and neck synovial sarcomas. J Surg oncol.

[B6] Akerman M, Willen H, Carlen B, Mandahl N, Mertens F (1996). Fine needle aspiration (FNA) of synovial sarcoma a comparative histological-cytological study of 15 cases, including immunohistochemical, electron microscopic and cytogenetic examination and DNA-ploidy analysis. Cytopathology.

[B7] Folpe AL, Schmidt RA, Chapman D, Gown AM (1998). Poorly differentiated synovial sarcoma: immunohistochemical distinction from primitive neuroectodermal tumors and high-grade malignant peripheral nerve sheath tumors. Am J Surg Pathol.

[B8] Klijanienko J, Caillaud JM, Lagace R, Vielh P (2002). Cytohistologic correlations in 56 synovial sarcomas in 36 patients: the Institut Curie experience. Diagn Cytopathol.

[B9] Luna-Ortiz K, Navarrete-Aleman JE, Granados-Garcia M, Herrera-Gomez A (2005). Primary parapharyngeal space tumors in a Mexican cancer center. Otolaryngol Head Neck Surg.

[B10] Sturgis EM, Potter BO (2003). Sarcomas of the head and neck region. Curr Opin Oncol.

[B11] Akerman M, Idvall I, Rydholm A (1980). Cytodiagnosis of soft tissue tumors and tumor like conditions by means of fine needle aspiration. Arch Orthop Trauma Surg.

[B12] Viguer JM, Jime'nez-Heffernan JA, Vicandi B, Lo'pez-Ferrer P, Gamallo C (1998). Cytologic features of synovial sarcoma with emphasis on the monophasic fibrous variant. A morphologic and immunocytochemical analysis of bcl-2 protein expression. Cancer (Cancer Cytopathol).

[B13] Ryan MR, Stastny JF, Wakely PE (1998). The cytopathology of synovial sarcoma. A study of six cases, with emphasis on architecture and histopathologic correlation. Cancer (Cancer Cytopathol).

[B14] Bertolini F, Bianchi B, Pizzigallo A, Tullio A, Sesenna E (2003). Synovial cell sarcoma of the neck. Case report and review of the literature. Acta Otorhinolaryngol Ital.

[B15] Lewis JJ, Antonescu CR, Leung D (2000). Synovial sarcoma: a multivariate analysis of prognostic factors in 112 patients with primary localized tumors of the extremity. Clin Oncol.

